# Phosphorylcholine is located in *Aggregatibacter actinomycetemcomitans* fimbrial protein Flp 1

**DOI:** 10.1007/s00430-018-0554-1

**Published:** 2018-07-28

**Authors:** Riikka Ihalin, Deyu Zhong, Maribasappa Karched, Casey Chen, Sirkka Asikainen

**Affiliations:** 1 Oral Microbiology, Institute of Dentistry, Umeå University, Umeå, Sweden; 2 Department of Biochemistry, University of Turku, Turku, Finland; 3 Department of Periodontics, Stomatological Hospital, Southern Medical University, Guangzhou, People’s Republic of China; 4 Department of Bioclinical Sciences, Faculty of Dentistry, Kuwait University, Kuwait City, Kuwait; 5 Herman Ostrow School of Dentistry, University of Southern California, Los Angeles, CA USA

**Keywords:** *Aggregatibacter actinomycetemcomitans*, Serotypes, Periodontitis, Phosphorylcholine, Fimbriae, Flp1, C-reactive protein, Serum resistance

## Abstract

Phosphorylcholine (ChoP) is covalently incorporated into bacterial surface structures, contributing to host mimicry and promoting adhesion to surfaces. Our aims were to determine the frequency of ChoP display among *Aggregatibacter actinomycetemcomitans* strains, to clarify which surface structures bear ChoP, and whether ChoP-positivity relates to serum killing. The tested oral (*N* = 67) and blood isolates (*N* = 27) represented 6 serotypes. Mab TEPC-15 was used for immunoblotting of cell lysates and fractions and for immunofluorescence microscopy of cell surface-bound ChoP. The lysates were denatured with urea for hidden ChoP or treated with proteinase K to test whether it binds to a protein. Three ChoP-positive and two ChoP-negative strains were subjected to serum killing in the presence/absence of CRP and using Ig-depleted serum as complement source. Cell lysates and the first soluble cellular fraction revealed a < 10 kDa band in immunoblots. Among 94 strains, 27 were ChoP positive. No difference was found in the prevalence of ChoP-positive oral (21/67) and blood (6/27) strains. Immunofluorescence microscopy corresponded to the immunoblot results. Proteinase K abolished ChoP reactivity, whereas urea did not change the negative result. The TEPC-15-reactive protein was undetectable in Δ*flp1* mutant strain. The survival rate of serotype-b strains in serum was 100% irrespective of ChoP, but that of serotype-a was higher in ChoP-positive (85%) than ChoP-negative (71%) strains. The results suggest that a third of rough-colony strains harbor ChoP and that ChoP is attached to fimbrial subunit protein Flp1. It further seems that ChoP-positivity does not enhance but may reduce *A. actinomycetemcomitans* susceptibility to serum killing.

## Introduction

Choline is a small water-soluble quaternary ammonium molecule and an essential nutrient for eukaryotes, including mammals [1]. Both eukaryotes and prokaryotes can express choline in the form of phosphorylated choline or phosphorylcholine (ChoP; phosphocholine, choline phosphate), which has a hydrophilic polar headgroup in some membrane lipids. While viable eukaryotic cells hide ChoP in the cell membrane, bacteria incorporate and expose it on cell surface proteins or glycoconjugates [2, 3].

Host mimicry through the ChoP moiety gives bacteria functional variability and helps them evade host immune recognition, thereby promoting survival in the host [4]. It enhances bacterial colonization on epithelial and endothelial cells through platelet activating factor receptor (PAFr) [5]. Among oral species, ChoP-positive *Aggregatibacter actinomycetemcomitans* strains use PAFr to adhere and enter human endothelial cells [6]. *Aggregatibacter actinomycetemcomitans* is associated with aggressive periodontitis and, similar to the other HACEK group bacteria, can cause serious non-oral infections such as infective endocarditis and abscesses in various parts of the body [7–9].

ChoP-displaying bacteria are common in the oropharyngeal and upper respiratory tracts. In a study by Gmur and coworkers, more than a third of tested dental plaque samples were positive for ChoP [10], while a fifth of examined oral species were ChoP-positive, among them *A. actinomycetemcomitans, A. aphrophilus, Fusobacterium nucleatum, Eikenella corrodens* and various *Actinomyces* and *Streptococcus* species. They also found intraspecies differences in ChoP positivity; only a third of *A. actinomycetemcomitans* and *Fusobacterium nucleatum* strains were ChoP positive, but the reason for strain-to-strain variability remained unknown.

Localization of ChoP in bacterial structures has been studied particularly in pathogens such as *Streptococcus pneumoniae, Haemophilus influenzae, Neisseria meningitidis* and *N. gonorrhoeae*, as well as nematodes [4, 11]. Interestingly, the localization of ChoP on the neisserial cell surface is related to the pathogenicity of the species; in commensal neisserial species the ChoP hapten is in lipooligosaccharide (LOS) [12], but in pathogenic *Neisseria*, it is covalently linked to a serine residue in fimbriae [3, 13]. The structures bearing ChoP are poorly known in gram-negative oral microbiota.

Extensive literature shows that ChoP has powerful immunomodulatory properties [14]. Despite the fact that mimicry of host molecules gives ChoP-bearing bacteria an advantage, it may also be unfavorable due to the specific binding of C-reactive protein (CRP) to ChoP and the presence of natural antibodies (IgM) against ChoP [15]. Through binding to ChoP, both CRP and IgM can activate the classical complement pathway leading to lysis of bacterial cells. Among oral species, resistance to serum killing has been reported in a few *A. actinomycetemcomitans* strains [16, 17], but no evidence has been found of the relationship between the ChoP display and sensitivity of *A. actinomycetemcomitans* to serum killing.

In this study, our aims were to determine the frequency of carrying ChoP among *A. actinomycetemcomitans* strains of different phenotypes, to clarify to which cell surface structures ChoP is attached and to determine whether ChoP affects the sensitivity of *A. actinomycetemcomitans* to CRP-mediated serum killing.

## Materials and methods

### Bacteria, culture conditions and phenotypic characteristics

The *A. actinomycetemcomitans* strains originated from S. Asikainen’s strain collection [18, 19], excluding the strains D7S, D7SS and D7S Δ*flp1* − *flp2*::Spe [20], or were purchased from ATCC (American Type Culture Collection, Manassas, Virginia, USA) or CCUG (Culture Collection, University of Gothenburg) culture collections. The strains CU1000R and CU1000S were a kind gift from J. B. Kaplan and D. H. Fine.

*Aggregatibacter actinomycetemcomitans* strains (67 oral and 27 blood isolates) were maintained at − 80 °C in 20% milk. The strains were revived on supplemented blood agar plates [5% defibrinated horse blood, 5 mg/l hemin (Sigma, St. Louis, MO, USA), 10 mg/l Vitamin K (Sigma), Columbia agar base (Acumedia, Baltimore, MD, USA)] and incubated in 5% CO_2_ in air at 37 °C for 3 days. For the immunoblot analysis of the D7S Δ*flp1* − *flp2*::Spe mutant and the D7S cell fractions, the mutant strain and D7S wild type control strain were revived on Tryptic soy agar (LabM, Lancashire, UK) plates supplemented with 5% defibrinated sheep blood.

The colonies of each *A. actinomycetemcomitans* strain were examined under a stereo-microscope (SMZ800, Nikon, Japan) using up to × 50 magnification. The colony morphology was defined as rough (wild type) when the colonies were circular, transparent and had a rough surface, often with a star-like inner structure and somewhat raised irregular edge [21]. The smooth colonies (spontaneous laboratory variants of wild type isolates) were generally larger than the rough colonies, were opaque and had a smooth surface and edges. The rough colonies were tightly adherent to the agar surface whereas the smooth colonies were easily removable with a loop.

The test strains for the present study were selected at random except for their serotype. *Aggregatibacter actinomycetemcomitans* serotypes represent different genetic lineages [22, 23]. Therefore, to ensure clonal diversity, the oral *A. actinomycetemcomitans* test strains were selected to represent 6 serotypes (a–f), with at least 10 strains for each serotype. Serotyping was carried out by immunodiffusion assay using serotype-specific antisera and/or PCR amplification of the serotype-specific sequences as previously described [24, 25].

### Immunoassays

ChoP-specific monoclonal mouse IgA antibody TEPC-15 (M1421, Sigma) was used for immunoblot analysis of *A. actinomycetemcomitans* cell lysates and for indirect immunofluorescence microscopy, which was set up to determine if the ChoP moiety is exposed on the bacterial cell surface.

SDS-PAGE and immunoblotting were performed as described previously [26] or with modifications as follows. Bacterial cells were washed with phosphate-buffered saline (PBS), pH 7.3, and incubated at 100 °C for 15 min in sample buffer [2% (w/v) SDS, 25% (v/v) glycerol, 0.1% (v/v) β-mercaptoethanol, 0.01% bromophenol blue (Merck, Darmstadt, Germany), 62.5 mM Tris HCl (pH 6.8)]. Samples with 15 µg protein [27] were separated on 8–16% (or 4–20% for Δ*flp1* testing) gradient gels (Criterion™, Bio-Rad, USA) in electrophoresis buffer [0.1% SDS, 192 mM glycine, 25 mM Tris (pH 8.3)] at constant voltage (150 V). For the immunoblot analysis, the proteins were transferred onto polyvinylidene fluoride membranes (Perkin Elmer, Boston, MA, USA) using a discontinuous buffer system [60 mM Tris and 40 mM CAPS, with 15% methanol (anode buffer) and 0.1% SDS (cathode buffer)] and constant current (140 mA) for 1 h (Trans-Blot^®^ SD Semi-Dry Transfer Cell, Bio-Rad, USA). After protein transfer, nonspecific reactivity was blocked with 5% (w/v) nonfat skim milk in TTBS [0.1% (v/v) Tween20, 500 mM NaCl, 20 mM Tris, pH 7.4] at 4 °C overnight and then incubated with 2–1 µg/ml dilution of MAb TEPC-15 (Sigma), depending on the lot, at RT for 1 h. The blots were then briefly washed and incubated at RT for 1 h with peroxidase-conjugated goat-anti-mouse IgA (Sigma) at a dilution of 50 ng/ml. After a final wash with TTBS, a reaction was detected by chemiluminescence substrate (SuperSignal^®^, Pierce, Rockford, IL, USA). Autoradiographic films (AGFA Cronex 5 Medical X-ray films, AGFA-Gevaert NV, Morsel, Belgium) exposed to the membranes were developed and scanned.

For indirect immunofluorescence microscopy, the bacterial cells were collected into 1% BSA in 0.01 M PBS to an optical density of 1 at 600 nm (1 × 10^9^ cells/ml). Then, 5 µl of each bacterial suspension was placed onto microscopic slides (Cel-Line, Novakemi AB, Enskede, Sweden) and air-dried. After adding TEPC-15 at a dilution of 6.3 µg/ml in BSA-PBS, the samples were incubated at RT for 1 h in a moist chamber. The slides were washed 10 min × 4 in PBS. FITC-conjugated goat anti-mouse IgA (Sigma) in BSA-PBS was added at a 13 µg/ml dilution and incubated at RT for 1 h in a moist chamber in the dark. After washing 10 min × 4 and 1 h × 2 in PBS in the dark, slides were observed using a fluorescence microscope (Leica DMRBE, FITC Exciter filter M2 No. 513,811, Leica Microscopes, Wetzlar, Germany) at × 1000 magnification. The specificity was demonstrated by the abolished TEPC-15 reactivity with *A. actinomycetemcomitans* when free ChoP (400 µg/ml) (Sigma) was added as a competitive inhibitor.

### Proteinase K treatment

To test whether the structural molecule in *A. actinomycetemcomitans* bearing the ChoP hapten was a protein, proteinase K was added (final concentration 2 mg/ml) to the bacterial sonicate (Branson Sonifier B-30^®^, Branson Sonic Power Co., USA) and the sample was incubated at 37 °C for 60 min prior to SDS-PAGE and immunoblot analysis as above.

### Urea treatment

To test whether the target structure would be hidden in TEPC-15 nonreactive *A. actinomycetemcomitans* strains, the whole-cell lysates were denatured by urea (final concentration 3 M, 100 °C, 5 min) prior to SDS-PAGE separation and immunoblotting as above.

### Purification of bacterial cell fractions

*Aggregatibacter actinomycetemcomitans* D7S cells were fractionated using a protocol for outer membrane protein (OMP) extraction [28]. Shortly, the cells harvested in HEPES buffer (10 mM HEPES, pH 7.4) were lysed by sonication (Branson Sonifier B-30 with Microtip, 5 × 1 min, Continuous duty cycle 50, Output control 4) and the intact cells and cell debris was removed by centrifugation (1700*g*, 20 min, 4 °C). The supernatant was then centrifuged (100,000*g*, 1 h, 4 °C) to collect the cell membranes. First sample which contained also the soluble loose fimbriae, was taken from the supernatant after the first ultracentrifugation and stored at − 80 °C. The cytoplasmic membrane was solubilized from the total membrane fraction in sodium lauryl sarcosinate (2% sodium lauryl sarcosinate, 10 mM HEPES, pH 7.4) and the insoluble fraction containing membrane-attached OMPs was collected by ultracentrifugation as described above. The OMP fraction was washed once with buffer A (1% octyl-β-d-glucopyranoside, 5 mM EDTA, 50 mM Tris–HCl, pH 8.0) and once with buffer A supplemented with 0.5 M NaCl, and the insoluble OMP fraction was collected by ultracentrifugation as above. Samples were taken from supernatants in each step and immunoblotted together with OMP sample using TEPC-15 antibody as described above.

### Serum bactericidal assay

The complement-mediated serum bactericidal assay was performed as described [29]. Briefly, three TEPC-15 positive and two TEPC-15 negative strains were tested. Cell suspensions (25 µl, 2 × 10^5^ cfu/ml) in Dulbecco’s complete PBS (61.5 µl) were pre-incubated with CRP (2.5 µl, 0.2 mg/ml; 5% CO_2_ in air, 37 °C, 20 min; Sigma) in the presence of CaCl_2_ (1 µl, 1 M). The mixtures were then incubated with Ig-depleted human serum (10 µl, 37 °C, 15 min; Sigma) as a source of complement. Control samples were otherwise similar except that serum was heat inactivated (56 °C, 30 min). After serial dilution, plating, and incubation as above, bacterial colonies were counted. To determine the percentage survival of bacteria, colony counts were compared between the results obtained using untreated and heat-inactivated serum.

All experiments were repeated at least twice.

### Statistics

Nonparametric tests were used to determine if the differences in frequency distributions were statistically significant; *P* < 0.05 was regarded significant.

## Results

### The reactivity to MAb TEPC-15 was strain-dependent

**Fig. 1:**
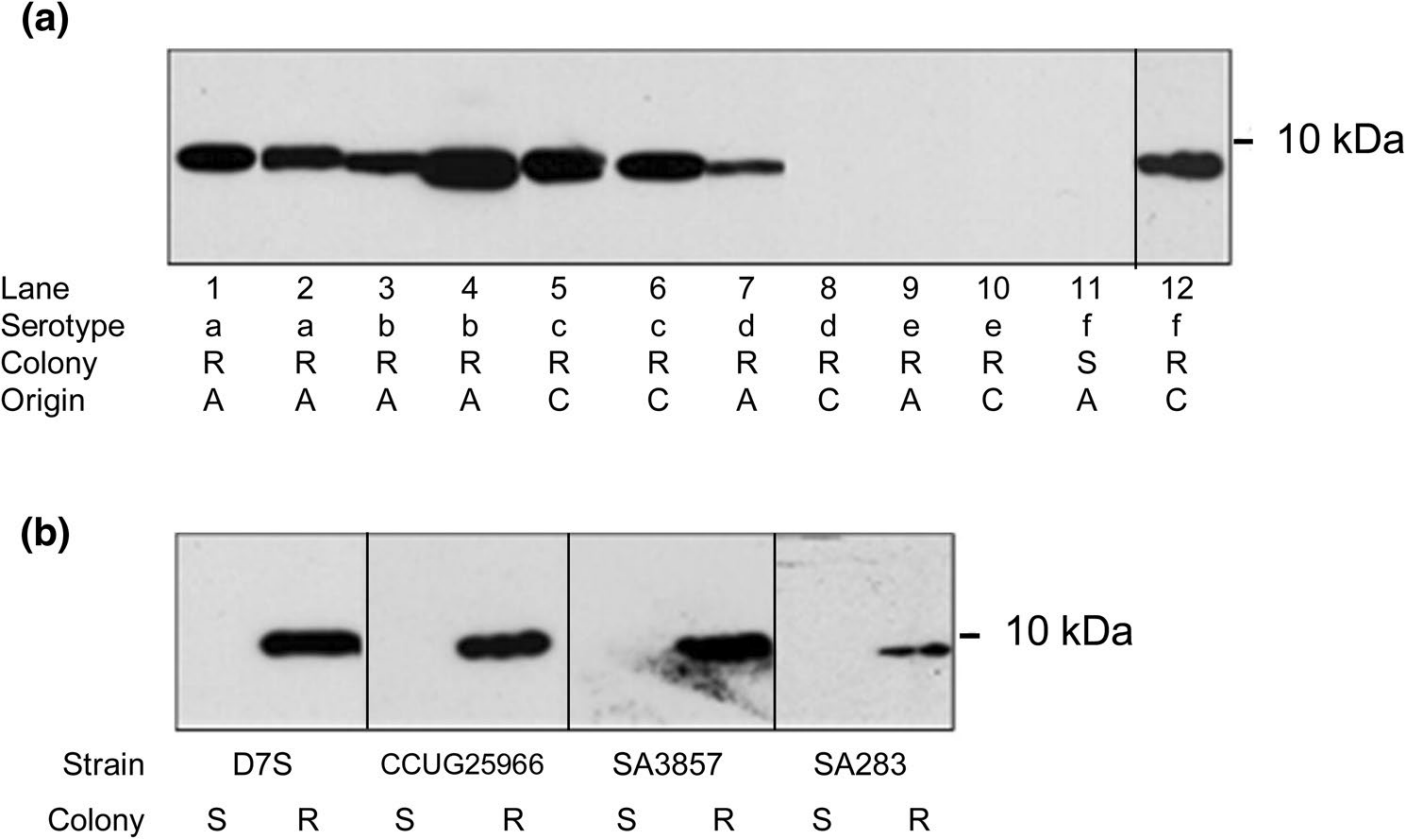
Reactivity of *A. actinomycetemcomitans* strains of six serotypes with Mab TEPC-15 using an immunoblot assay. Serotype (a–f), rough (R) or smooth (S) colony morphology and origin of the strains from patients with aggressive (A) or chronic (C) periodontitis are shown for each strain separately. Strain identification by lane number: (1) SA558, (2) D7S, (3) SA716, (4) SA1398, (5) SA3857, (6) SA1251, (7) SA3710, (8) SA3033, (9) SA1350, (10) SA1655, (11) CU1000, and (12) SA3632 **(a)**. *Aggregatibacter actinomycetemcomitans* colony morphology in relation to reactivity with TEPC-15 antibody. Immunoblot with whole-cell lysates from spontaneous smooth-colony variants (S) of the rough-colony strains (R) **(b)**

*A. actinomycetemcomitans* test strains represented 6 serotypes (a-) to ensure their clonal diversity. Immunoblot analysis of the cell lysates using TEPC-15 antibody demonstrated a single < 10 kDa band in 27 of 94 strains (28.7%) (Table 1; Fig. 1a). Urea treatment of the lysates to expose hidden ChoP did not change the negative immunoblot result (not shown).

**Table 1:** Colony morphology-dependence of Mab TEPC-15 reactivity with *A. actinomycetemcomitans* whole-cell lysates

Reactivity with Mab TEPC-15	All test strains		
	Smooth *N* (%)	Rough *N* (%)	Total *N* (%)
Positive	0 (0)	27 (34.2)	27 (28.7)
Negative	15 (100)	52 (65.8)	67 (71.3)
Total	15 (100%)	79 (100%)	94 (100%)

*P* < 0.007, Chi-Square, *df* 1

The TEPC-15 positivity significantly differed between serotypes, either when all 94 *A. actinomycetemcomitans* strains (*P* = 0.008, Chi-Square, *df* = 5) or only rough-colony strains (*P* = 0.001, Chi-Square, *df* = 5) were analyzed (Table 2). Serotype-a was most frequently TEPC-15 positive (79% of the rough-colony strains), whereas none of the 10 serotype-e strains tested were positive.

**Table 2:** Serotype-dependence of Mab TEPC-15 reactivity with *A. actinomycetemcomitans* whole-cell lysates

Serotype	All test strains	Rough-colony strains
	No. positive strains/no. strains tested (%)	No. positive strains/no. strains tested (%)
a	11/18 (61.1)	11/14 (78.6)
b	8/27 (29.6)	8/20 (40.0)
c	4/15 (26.7)	4/13 (30.8)
d	1/11 (9.1)	1/11 (9.1)
e	0/10 (0)	0/10 (0)
f	3/13 (23.1)	3/11 (27.3)
Total	27/94 (28.7%)	27/79 (34.2%)

All strains: *P* < 0.008, Chi-Square, *df* = 5; Rough-colony strains: *P* < 0.001 Chi-Square, *df* = 5

We next analyzed the relationship between TEPC-15 reactivity and the origin of *A. actinomycetemcomitans* strains from either the oral cavity or venous blood. The results of the rough-colony strains showed no significant difference between oral (21/60, 35%) and blood strains (6/19, 32%) (*P* = 0.78, Chi-Square, *df* = 1) (Table 3).

**Table 3:** Dependence of Mab TEPC-15 reactivity on isolation origin and colony morphology of *A. actinomycetemcomitans* strains

Reactivity with Mab TEPC-15	All test strains			Rough-colony strains		
	Oral *N* (%)	Blood *N* (%)	Total *N* (%)	Oral *N* (%)	Blood *N* (%)	Total *N* (%)
Positive	21 (31.3)	6 (22.2)	27 (28.7)	21 (35.0)	6 (31.6)	27 (34.2)
Negative	46 (68.7)	21 (77.8)	67 (71.3)	39 (65.0)	13 (68.4)	52 (65.8)
Total	67 (100%)	27 (100%)	94 (100%)	60 (100%)	19 (100%)	79 (100%)

All strains *P* = 0.337, Chi-Square, *df* = 1; Rough-colony strains *P* = 0.784, Chi-Square, *df* = 1

The oral strains were divided into groups according to the periodontal status of the donor (Table 4). No significant difference (*P* = 0.211, Chi-Square, *df* = 3) was seen in the TEPC-15 reactivity and periodontal status group. The difference was also not significant (*P* = 0.330, Chi-Square, *df* = 1) when only strains from aggressive periodontitis and chronic periodontitis were included in the analysis.

**Table 4:** Frequency distribution of immunoblot reactivity with Mab TEPC-15 among *A. actinomycetemcomitans* whole-cell lysates obtained from patients with differing periodontal status

Periodontal status	Mab TEPC-15+ *N* (%)	Mab TEPC-15 – *N* (%)	Total *N* (%)
Aggressive periodontitis	11 (37.9)	18 (62.1)	29 (100)
Chronic periodontitis	9 (26.5)	25 (73.5)	34 (100)
Gingivitis	1 (100)	0	1 (100)
Healthy	0	3 (100)	3 (100)
Total (*N*)	21	46	67

*P* = 0.211, Chi-Square, *df* = 3

The reactivity of *A. actinomycetemcomitans* strains with TEPC-15 was significantly (*P* = 0.007, Chi-Square, *df* = 1) associated with the colony morphology: 27/79 (34.2%) of rough-colony strains but none of the 15 smooth-colony strains were TEPC-15 immunoblot positive (Table 1). To further test the relationship between TEPC-15 reactivity and *A. actinomycetemcomitans* colony morphology, we compared four of the spontaneous smooth-colony variants with their respective rough-colony counterparts. All four smooth-colony variants were nonreactive, whereas all respective rough-colony strains reacted with TEPC-15 (Fig. 1b).

### The protein bearing ChoP was surface-exposed on *A. actinomycetemcomitans* cells

**Fig. 2:**
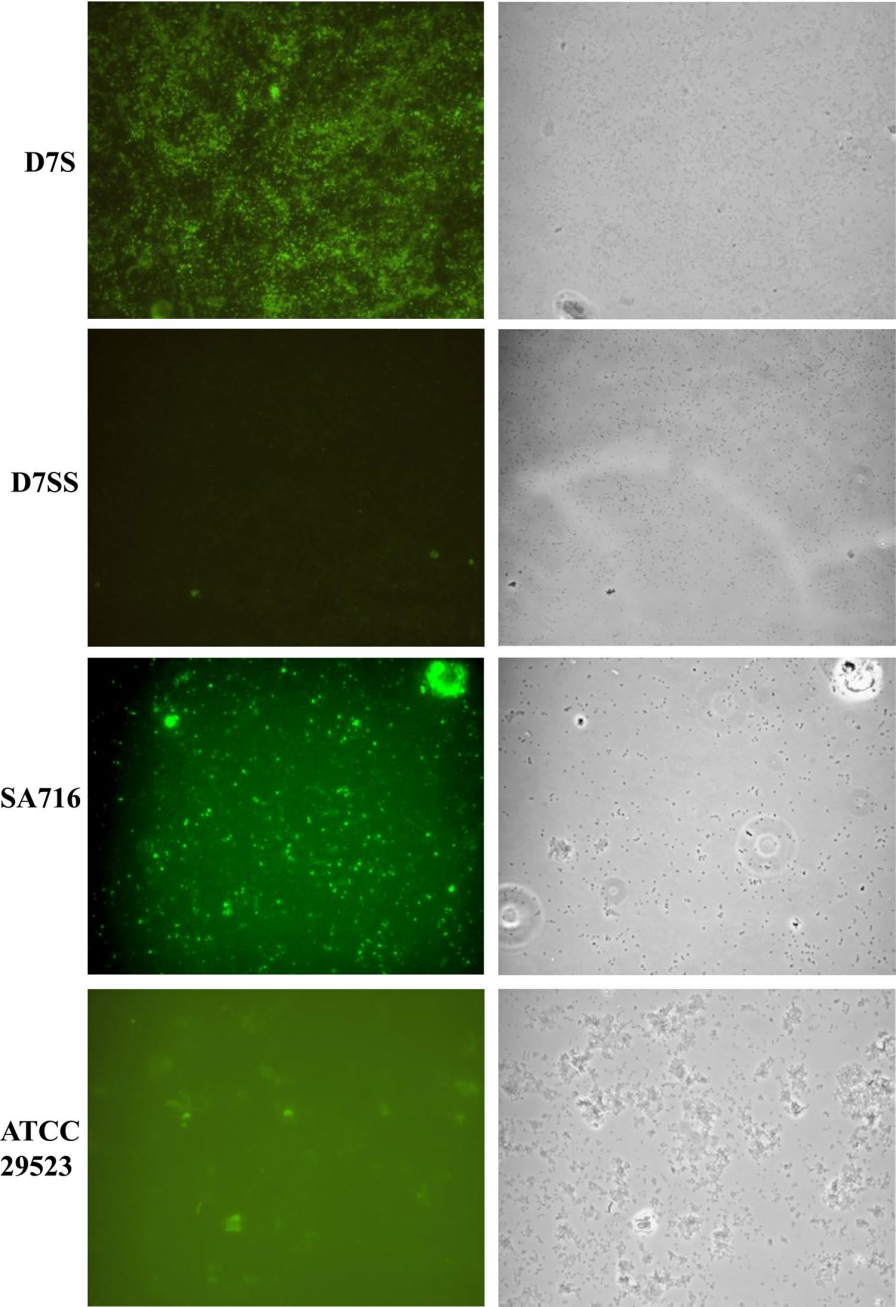
Reactivity of rough-colony (D7S, SA716) and smooth-colony (D7SS, ATCC 29523) *A. actinomycetemcomitans* strains with Mab TEPC-15 using indirect immunofluorescence microscopy and phase contrast microscopy in parallel. Original magnification ×1000

To confirm the accessibility of TEPC-15 to ChoP, immunofluorescence analysis of *A. actinomycetemcomitans* cells was performed. The results were in accordance with those obtained by immunoblot assays and indicated that the ChoP hapten was surface-exposed (Fig. 2).

### ChoP was located in Flp1

**Fig. 3:**
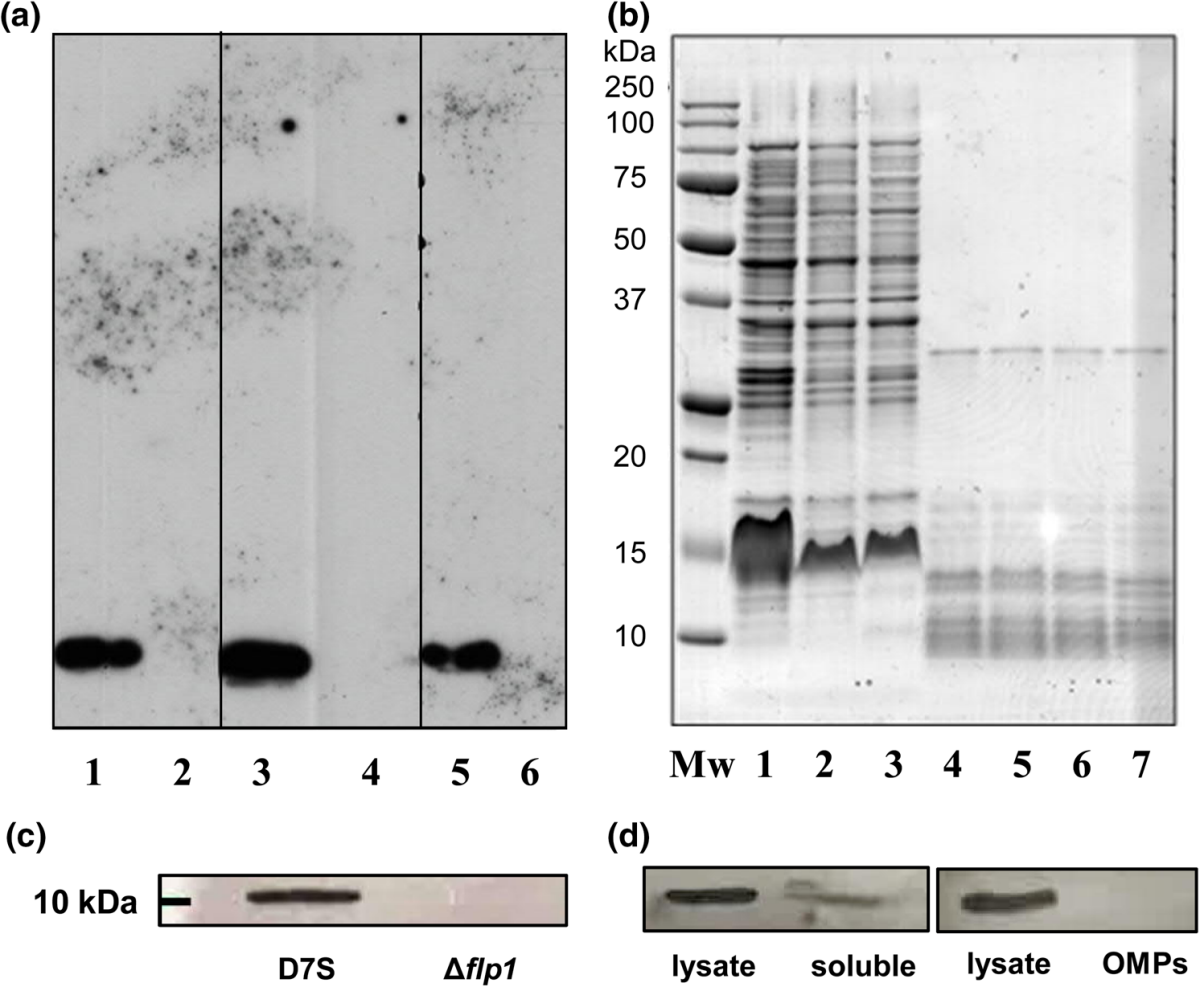
The Mab TEPC-15 reactive protein was the surface-exposed protein Flp1. Effect of proteinase K treatment on the immunoblot reactivity of *A. actinomycetemcomitans* whole-cell lysates with Mab TEPC-15. *Aggregatibacter actinomycetemcomitans* strains SA1398, SA716 and D7S without (lanes 1, 3, 5) and with (lanes 2, 4, 6) proteinase K treatment (**a**). Coomassie blue staining of the SDS-PAGE gel before (lanes 1–3) and after (lanes 4–6) proteinase K treatment. Lane 7: proteinase K in PBS (the molecular weight of proteinase K from Sigma is 28.93 kDa). MW: molecular weight standard (**b**). The Δ*flp1* mutant did not react with Mab TEPC-15 in contrast to the parental wild type D7S (**c**). From OMP extracts of the wild type strain D7S, only the first soluble protein fraction after cell lysis reacted with Mab TEPC-15, similarly to the D7S whole-cell lysate (**d**)

To examine whether the bacterial structure bearing ChoP was a protein, the cell lysates of TEPC-15 positive *A. actinomycetemcomitans* strains were treated with proteinase K. Proteinase K treatment abolished TEPC-15 reactivity of *A. actinomycetemcomitans* lysates (Fig. 3a). After Coomassie blue staining for proteins, the samples without proteinase K treatment showed a variety of bands distributed from low to high molecular weight area (Fig. 3b). A proteinase K band (29 kDa) was visible in all samples to which it was added (Fig. 3b).

Earlier studies have shown that Flp fimbriae are responsible for the rough-colony phenotype of *A. actinomycetemcomitans* [30]. Our findings demonstrated that none of the smooth variants was TEPC-15 positive and that, in rough-colony strains reacting with TEPC-15, the molecular mass of the found protein was similar to that of the fimbrial subunit Flp1. Therefore, we next tested the TEPC-15 reactivity of the Δ*flp1* mutant. The immunoblot showed no reactivity of the Δ*flp1* mutant to the TEPC-15, whereas the parental wild type strain D7S reacted at a size of approximately 10 kDa (Fig. 3c).

To verify the results obtained from immunofluorescence analysis which suggested that the molecule bearing ChoP was surface exposed, we immunoblotted various *A. actinomycetemcomitans* D7S cell fractions with TEPC-15 antibody. Only the first soluble fraction, excluding the inner membrane and outer membrane proteins, showed reactivity to TEPC-15 similar to the D7S whole-cell lysate, whereas no reactivity was found in the OMP fraction (Fig. 3d).

### Reactivity with Mab TEPC-15 did not increase the susceptibility of *A. actinomycetemcomitans* to CRP-mediated complement killing

**Fig. 4:**
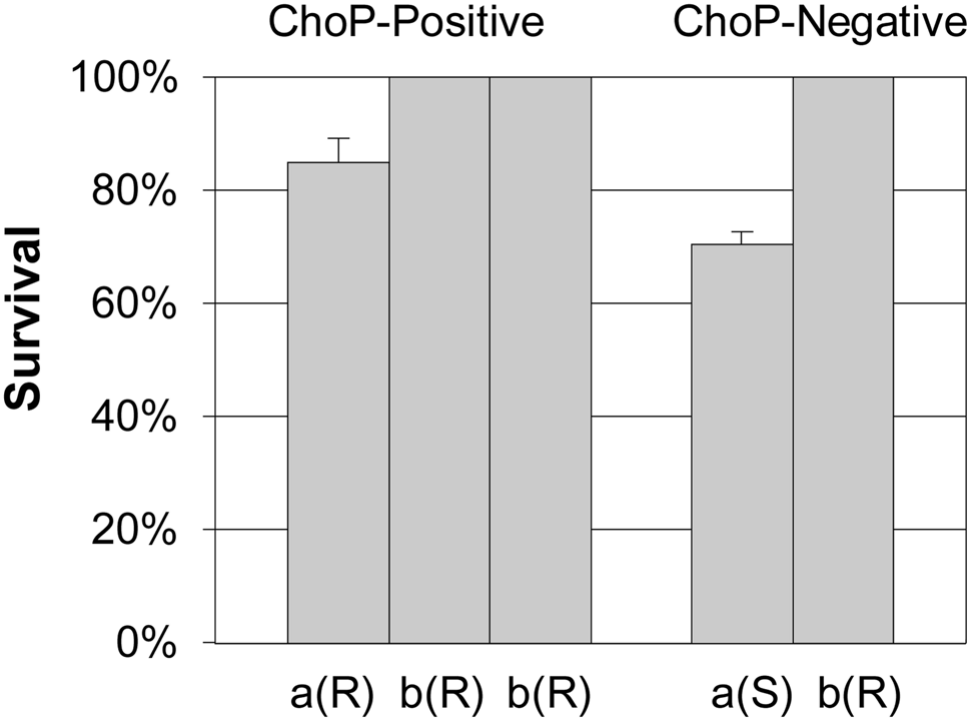
Sensitivity of ChoP-positive and ChoP-negative *A. actinomycetemcomitans* strains to serum killing mediated by CRP. The percentage survival represents the number of CFU in the presence of untreated serum compared to controls containing heat-inactivated serum. ChoP-positive and ChoP-negative strains, their serotypes (a/b) and colony morphology (rough (R) or smooth (S)) are shown separately. Strain identification from left to right: D7S, SA716, SA1398, D7SS, SA1065

A total of five *A. actinomycetemcomitans* strains of serotypes a and b were selected as test strains. The serotype-a strains included a rough-colony strain (D7S), reactive with TEPC-15, and its smooth-colony variant (D7SS), nonreactive with TEPC-15. Two (SA716 and SA1398) serotype-b strains with rough-colony phenotypes reacted with TEPC-15 and one (SA1065) was nonreactive with TEPC-15. All three serotype-b strains, irrespective of their reactivity with TEPC-15, showed complete resistance to CRP-mediated complement killing (Fig. 4). Both phenotypes of the serotype-a strain were relatively resistant to serum, with a mean survival rate of D7S being higher (85% ± 0.04) than that of D7SS (71% ± 0.02).

## Discussion

This study demonstrated that the reactivity of *A. actinomycetemcomitans* with the ChoP-specific MAb TEPC-15 was related to the specific strains’ rough colony morphology and serotype but not to the origin of the strains, being either oral cavity, venous blood or the type of periodontitis. All smooth-colony strains were ChoP negative and all ChoP-positive strains formed rough colonies. When ChoP-positive rough-colony strains and their spontaneous smooth-colony variants were available for testing in parallel, the result remained the same, with the smooth-colony variants always appearing ChoP negative. In all ChoP-positive strains, an immunoblot of the cellular lysate revealed a single band of < 10 kDa molecular weight. Proteinase K treatment of cellular lysates abolished the reactivity, giving evidence for presence of ChoP in association with a protein. Immunofluorescence microscopy showed that ChoP was on the cell surface, accessible to the TEPC-15 antibody. Moreover, the mutant lacking *flp1* gene and *flp2* pseudogene [20], unable to produce fimbriae consisting of Flp1 pili subunits, did not react with the TEPC-15 antibody. From the tested *A. actinomycetemcomitans* cell fractions only the first soluble protein fraction after cell lysis reacted with TEPC-15. In the fimbriae extraction protocol, the fimbriae are precipitated from the first soluble protein fraction after bacterial cell homogenization [31], suggesting that, although located in the bacterial extracellular space, the fimbriae are not attached to the outer membrane with such a high affinity which would enable them to be extracted together with OMPs. It has long been known that, contrary to the smooth-colony variants, the *A. actinomycetemcomitans* rough-colony phenotype, the wild type, invariably expresses long, bundled fimbriae [20, 31–33]. Thus, our results strongly suggest that in *A. actinomycetemcomitans* ChoP is surface-exposed and located on Flp1 fimbriae. A potential amino acid residue where ChoP could be covalently attached is serine, as earlier shown in *N. gonorrhoeae* [13].

Still, the mere presence of fimbriae cannot be the sole determinant for bearing ChoP, since two-thirds of the present rough-colony strains did not react with the TEPC-15 antibody. Similar findings were reported in *N. meningitidis* in which 60% of the test strains were TEPC-15 negative in immunoblot [3]. Also similar to the *A. actinomycetemcomitans* Flp1 fimbriae, *N. meningitidis* displays fimbriae consisting of PilE pili subunits, which belong to the type IV pili, and TEPC-15 reacts with fimbriated *N. meningitidis* strains only. However, the PilE of *N. meningitidis* belongs to the type IVa pili, whereas the Flp1 of *A. actinomycetemcomitans* belongs to the type IVb pili [32]. A previous finding of a single TEPC-15 reactive band the size of the fimbrillin subunit protein of *N. meningitidis* corresponds to our results demonstrating a single TEPC-15 positive band of *A. actinomycetemcomitans*. However, the size of our TEPC 15-positive band differed from the size of unmodified mature Flp1, which is approximately 5.1 kDa. The size difference could be explained by the fact that *A. actinomycetemcomitans* Flp1 fimbrillin is glycosylated, therefore, increasing the molecular mass of Flp1 to approximately 6.2–6.5 kDa [34]. Moreover, the mature Flp1 monomers interact strongly with each other, making it difficult to disrupt the multimers even by boiling with SDS [35]. Thus, the TEPC-15 positive band of D7S in Fig. 3c, which had a size of approximately 10 kDa, could have originated from a mature Flp1 dimer [34].

To better understand the reason for finding TEPC-15 negative rough-colony strains, we examined the possibility that the ChoP hapten was hidden in cell surface structures. However, no change was seen in the reactivity of our strains, in contrast to *N. meningitidis* strains which, after urea treatment, displayed the ChoP moiety [3]. Another possibility is the phase variability of the ChoP display in fimbriae as has been shown in *N. meningitidis* [3, 36]. Phase variation of ChoP display could partly explain the differences in the reactivity of our rough-colony *A. actinomycetemcomitans* strains to the TEPC-15 antibody, but evidence for this hypothesis is yet to be attained.

Our results suggesting ChoP in a protein structure of *A. actinomycetemcomitans* differs from the previous report suggesting an association of ChoP with *A. actinomycetemcomitans* LPS [37]. We tested purified LPS preparations of some strains that were ChoP positive in immunoblot, but they were determined to be ChoP negative (not shown). In our preliminary genetic comparison, gene homologs of *licA* encoding phosphorylcholine kinase, which catalyzes the incorporation of ChoP into LPS in *H. influenzae*, were not found in *A. actinomycetemcomitans*. The *lic1* locus encodes phase variation of ChoP in *H. influenzae* LPS and commensal *Neisseria* species [4, 29, 38]. However, similar to *A. actinomycetemcomitans*, the *lic* gene family is absent in the pathogenic species *N. meningitidis* and *N. gonorrhoeae* [12]. Interestingly, a homolog(s) to *pptA*, encoding pilin phosphorylcholine transferase A to add ChoP to *N. meningitidis* fimbriae [36], was found in *A. actinomycetemcomitans* (strain D7S, genes D7S_01692 and D7S_02283).

The frequency distribution of the ChoP-positive *A. actinomycetemcomitans* strains differed between the serotype groups. A minimum of 10 random oral strains were analyzed from each of the 6 serotypes. When only rough-colony strains were included, it appeared that the prevalence of ChoP-positive strains was highest among serotype-a strains and lowest in serotype-d and serotype-e strains. While keeping in mind the limited number of present test strains in each serotype, the ChoP display among *A. actinomycetemcomitans* strains does not seem to be related to the evolutionary lineages of *A. actinomycetemcomitans*; serotypes a, d, e, and f are closer relatives with each other than with serotypes b and c [39]. On the other hand, the majority (81%) of the present ChoP-bearing oral *A. actinomycetemcomitans* strains belonged to serotypes a, b, and c, which are the most common serotypes of oral *A. actinomycetemcomitans* isolates found in a variety of geographically and ethnically diverse study populations [18, 24, 40–42]. This suggests that ChoP display may enhance the oral colonization of certain *A. actinomycetemcomitans* serotypes similarly to other common oropharyngeal species [10], although the mechanism remains unknown.

In this study, ChoP-positive strains were found almost equally among the oral and blood strains when only the rough-colony strains were compared. Finding a similar frequency of ChoP-positive strains in the oral cavity and blood was unexpected since ChoP renders the bacteria sensitive to serum killing [14]. For the present serum killing experiment we chose ChoP-positive and ChoP-negative strains of serotype-a and serotype-b and the CRP concentration (5 µg/ml) within the range observed in periodontitis patients’ blood [43]. We found no ChoP display-related difference in the survival of *A. actinomycetemcomitans* serotype-b strains. That the ChoP-positive serotype-a strain showed 17% higher survival rate than its ChoP-negative counterpart is interesting and renders further studies on possible serotype differences in the susceptibility to serum killing.

In summary, our study brings new knowledge to this topic: The results suggest that the occurrence rate of ChoP-positive *A. actinomycetemcomitans* strains depends on serotype. This apparently is inconsistent with their evolutionary lineages but rather consistent with their global prevalence in the oral cavity. Strong experimental evidence also indicates that the ChoP molecule is located on the fimbrial subunit protein Flp1. Furthermore, it seems that the ability of specified *A. actinomycetemcomitans* phenotypes to escape CRP-mediated serum killing is not affected by the presence or absence of ChoP, instead, the ChoP-positivity may relate to an enhanced survival rate of some *A. actinomycetemcomitans* phenotypes.

## References

[1] Zeisel SH, da Costa KA (2009). Choline: an essential nutrient for public health. Nutr Rev.

[2] Lysenko E, Richards JC, Cox AD, Stewart A, Martin A, Kapoor M, Weiser JN (2000). The position of phosphorylcholine on the lipopolysaccharide of *Haemophilus influenzae* affects binding and sensitivity to C-reactive protein-mediated killing. Mol Microbiol.

[3] Weiser JN, Goldberg JB, Pan N, Wilson L, Virji M (1998). The phosphorylcholine epitope undergoes phase variation on a 43-kilodalton protein in *Pseudomonas aeruginosa* and on pili of *Neisseria meningitidis* and *Neisseria gonorrhoeae*. Infect Immun.

[4] Clark SE, Weiser JN (2013). Microbial modulation of host immunity with the small molecule phosphorylcholine. Infect Immun.

[5] Cundell DR, Gerard NP, Gerard C, Idanpaan-Heikkila I, Tuomanen EI (1995). *Streptococcus pneumoniae* anchor to activated human cells by the receptor for platelet-activating factor. Nature.

[6] Schenkein HA, Barbour SE, Berry CR, Kipps B, Tew JG (2000). Invasion of human vascular endothelial cells by *Actinobacillus actinomycetemcomitans* via the receptor for platelet-activating factor. Infect Immun.

[7] Paturel L, Casalta JP, Habib G, Nezri M, Raoult D (2004). *Actinobacillus actinomycetemcomitans* endocarditis. Clin Microbiol Infect.

[8] Rahamat-Langendoen JC, van Vonderen MG, Engstrom LJ, Manson WL, van Winkelhoff AJ, Mooi-Kokenberg EA (2011). Brain abscess associated with *Aggregatibacter actinomycetemcomitans*: case report and review of literature. J Clin Periodontol.

[9] Paju S, Carlson P, Jousimies-Somer H, Asikainen S (2000). Heterogeneity of *Actinobacillus actinomycetemcomitans* strains in various human infections and relationships between serotype, genotype, and antimicrobial susceptibility. J Clin Microbiol.

[10] Gmur R, Thurnheer T, Guggenheim B (1999). Dominant cross-reactive antibodies generated during the response to a variety of oral bacterial species detect phosphorylcholine. J Dent Res.

[11] Harnett W, Rzepecka J, Houston KM (2010). How do nematodes transfer phosphorylcholine to carbohydrates?. Trends Parasitol.

[12] Serino L, Virji M (2000). Phosphorylcholine decoration of lipopolysaccharide differentiates commensal *Neisseriae* from pathogenic strains: identification of licA-type genes in commensal *Neisseriae*. Mol Microbiol.

[13] Hegge FT, Hitchen PG, Aas FE, Kristiansen H, Lovold C, Egge-Jacobsen W, Panico M, Leong WY, Bull V, Virji M, Morris HR, Dell A, Koomey M (2004). Unique modifications with phosphocholine and phosphoethanolamine define alternate antigenic forms of *Neisseria gonorrhoeae* type IV pili. Proc Natl Acad Sci USA.

[14] Clark SE, Snow J, Li J, Zola TA, Weiser JN (2012). Phosphorylcholine allows for evasion of bactericidal antibody by *Haemophilus influenzae*. PLoS Pathog.

[15] Volanakis JE (2001). Human C-reactive protein: expression, structure, and function. Mol Immunol.

[16] Sundqvist G, Johansson E (1982). Bactericidal effect of pooled human serum on *Bacteroides melaninogenicus, Bacteroides asaccharolyticus* and *Actinobacillus actinomycetemcomitans*. Scand J Dent Res.

[17] Asakawa R, Komatsuzawa H, Kawai T, Yamada S, Goncalves RB, Izumi S, Fujiwara T, Nakano Y, Suzuki N, Uchida Y, Ouhara K, Shiba H, Taubman MA, Kurihara H, Sugai M (2003). Outer membrane protein 100, a versatile virulence factor of *Actinobacillus actinomycetemcomitans*. Mol Microbiol.

[18] Asikainen S, Lai CH, Alaluusua S, Slots J (1991). Distribution of *Actinobacillus actinomycetemcomitans* serotypes in periodontal health and disease. Oral Microbiol Immunol.

[19] Dogan B, Saarela MH, Jousimies-Somer H, Alaluusua S, Asikainen S (1999). *Actinobacillus actinomycetemcomitans* serotype e–biotypes, genetic diversity and distribution in relation to periodontal status. Oral Microbiol Immunol.

[20] Wang Y, Chen C (2005). Mutation analysis of the flp operon in *Actinobacillus actinomycetemcomitans*. Gene.

[21] Karched M, Paul-Satyaseela M, Asikainen S (2007). A simple viability-maintaining method produces homogenic cell suspensions of autoaggregating wild-type *Actinobacillus actinomycetemcomitans*. J Microbiol Methods.

[22] Asikainen S, Chen C, Slots J (1995). *Actinobacillus actinomycetemcomitans* genotypes in relation to serotypes and periodontal status. Oral Microbiol Immunol.

[23] Kittichotirat W, Bumgarner RE, Asikainen S, Chen C (2011). Identification of the pangenome and its components in 14 distinct *Aggregatibacter actinomycetemcomitans* strains by comparative genomic analysis. PLoS One.

[24] Saarela M, Asikainen S, Alaluusua S, Pyhälä L, Lai CH, Jousimies-Somer H (1992). Frequency and stability of mono- or poly-infection by *Actinobacillus actinomycetemcomitans* serotypes a, b, c, d or e. Oral Microbiol Immunol.

[25] Dogan B, Antinheimo J, Cetiner D, Bodur A, Emingil G, Buduneli E, Uygur C, Firatli E, Lakio L, Asikainen S (2003). Subgingival microflora in Turkish patients with periodontitis. J Periodontol.

[26] Paul-Satyaseela M, Karched M, Bian Z, Ihalin R, Boren T, Arnqvist A, Chen C, Asikainen S (2006). Immunoproteomics of *Actinobacillus actinomycetemcomitans* outer-membrane proteins reveal a highly immunoreactive peptidoglycan-associated lipoprotein. J Med Microbiol.

[27] Lowry OH, Rosebrough NJ, Farr AL, Randall RJ (1951). Protein measurement with the Folin phenol reagent. J Biol Chem.

[28] Wilson ME (1991). The heat-modifiable outer membrane protein of *Actinobacillus actinomycetemcomitans*: relationship to OmpA proteins. Infect Immun.

[29] Serino L, Virji M (2002). Genetic and functional analysis of the phosphorylcholine moiety of commensal *Neisseria* lipopolysaccharide. Mol Microbiol.

[30] Wang Y, Liu A, Chen C (2005). Genetic basis for conversion of rough-to-smooth colony morphology in *Actinobacillus actinomycetemcomitans*. Infect Immun.

[31] Inoue T, Tanimoto I, Ohta H, Kato K, Murayama Y, Fukui K (1998). Molecular characterization of low-molecular-weight component protein, Flp, in *Actinobacillus actinomycetemcomitans* fimbriae. Microbiol Immunol.

[32] Kachlany SC, Planet PJ, Desalle R, Fine DH, Figurski DH, Kaplan JB (2001). *flp-1*, the first representative of a new pilin gene subfamily, is required for non-specific adherence of *Actinobacillus actinomycetemcomitans*. Mol Microbiol.

[33] Rosan B, Slots J, Lamont RJ, Listgarten MA, Nelson GM (1988). *Actinobacillus actinomycetemcomitans* fimbriae. Oral Microbiol Immunol.

[34] Inoue T, Ohta H, Tanimoto I, Shingaki R, Fukui K (2000). Heterogeneous post-translational modification of *Actinobacillus actinomycetemcomitans* fimbrillin. Microbiol Immunol.

[35] Tomich M, Fine DH, Figurski DH (2006). The TadV protein of *Actinobacillus actinomycetemcomitans* is a novel aspartic acid prepilin peptidase required for maturation of the Flp1 pilin and TadE and TadF pseudopilins. J Bacteriol.

[36] Warren MJ, Jennings MP (2003). Identification and characterization of *pptA*: a gene involved in the phase-variable expression of phosphorylcholine on pili of *Neisseria meningitidis*. Infect Immun.

[37] Schenkein HA, Gunsolley JC, Best AM, Harrison MT, Hahn CL, Wu J, Tew JG (1999). Antiphosphorylcholine antibody levels are elevated in humans with periodontal diseases. Infect Immun.

[38] Humphries HE, High NJ (2002). The role of *licA* phase variation in the pathogenesis of invasive disease by *Haemophilus influenzae* type b. FEMS Immunol Med Microbiol.

[39] Kittichotirat W, Bumgarner RE, Chen C (2016). Evolutionary Divergence of *Aggregatibacter actinomycetemcomitans*. J Dent Res.

[40] Chen C, Wang T, Chen W (2010). Occurrence of *Aggregatibacter actinomycetemcomitans* serotypes in subgingival plaque from United States subjects. Mol Oral Microbiol.

[41] Mombelli A, Gmur R, Lang NP, Corbert E, Frey J (1999). *Actinobacillus actinomycetemcomitans* in Chinese adults. Serotype distribution and analysis of the leukotoxin gene promoter locus. J Clin Periodontol.

[42] Zambon JJ, Umemoto T, De Nardin E, Nakazawa F, Christersson LA, Genco RJ (1988). *Actinobacillus actinomycetemcomitans* in the pathogenesis of human periodontal disease. Adv Dent Res.

[43] Mattila K, Vesanen M, Valtonen V, Nieminen M, Palosuo T, Rasi V, Asikainen S (2002). Effect of treating periodontitis on C-reactive protein levels: a pilot study. BMC Infect Dis.

